# Evaluating a selective prevention programme for binge drinking among young adolescents: study protocol of a randomized controlled trial

**DOI:** 10.1186/1471-2458-11-126

**Published:** 2011-02-21

**Authors:** Jeroen Lammers, Ferry Goossens, Suzanne Lokman, Karin Monshouwer, Lex Lemmers, Patricia Conrod, Reinout Wiers, Rutger Engels, Marloes Kleinjan

**Affiliations:** 1 Trimbos Institute (Netherlands Institute of Mental Health and Addiction), Utrecht, The Netherlands; 2 Department of Psychological Medicine and Psychiatry, Section of Addiction, Kings College London, University of London, UK; 3 Department of Psychology, University of Amsterdam, The Netherlands; 4 Behavioural Science Institute, Radboud University Nijmegen, The Netherlands

## Abstract

**Background:**

In comparison to other Europe countries, Dutch adolescents are at the top in drinking frequency and binge drinking. A total of 75% of the Dutch 12 to 16 year olds who drink alcohol also engage in binge drinking. A prevention programme called Preventure was developed in Canada to prevent adolescents from binge drinking. This article describes a study that aims to assess the effects of this selective school-based prevention programme in the Netherlands.

**Methods:**

A randomized controlled trial is being conducted among 13 to 15-year-old adolescents in secondary schools. Schools were randomly assigned to the intervention and control conditions. The intervention condition consisted of two 90 minute group sessions, carried out at the participants' schools and provided by a qualified counsellor and a co-facilitator. The intervention targeted young adolescents who demonstrated personality risk for alcohol abuse. The group sessions were adapted to four personality profiles. The control condition received no further intervention above the standard substance use education sessions provided in the Dutch national curriculum. The primary outcomes will be the percentage reduction in binge drinking, weekly drinking and drinking-related problems after three specified time periods. A screening survey collected data by means of an Internet questionnaire. Students have completed, or will complete, a post-treatment survey after 2, 6, and 12 months, also by means of an online questionnaire.

**Discussion:**

This study protocol presents the design and current implementation of a randomized controlled trial to evaluate the effectiveness of a selective alcohol prevention programme. We expect that a significantly lower number of adolescents will binge drink, drink weekly, and have drinking-related problems in the intervention condition compared to the control condition, as a result of this intervention.

**Trial registration:**

This trial is registered in the Dutch Trial Register (Cochrane Collaboration) as NTR1920.

## Background

Binge drinking is an increasing problem among young adolescents in the Netherlands. The recent use of alcohol among pupils in secondary education (12 to 16 years of age) in the Netherlands is declining, while binge drinking among these pupils is increasing. Nowadays, 75% of the Dutch 12 to 16 year olds who drink alcohol also engage in binge drinking [1]; this implies consuming five or more alcoholic drinks on one occasion in the past month. The largest proportion of binge drinkers are found in the age category of 15 and 16 years old. In comparison to other European countries, Dutch adolescents are among the leaders in drinking frequency and binge drinking [2,3].

In adolescents, heavy alcohol consumption is associated with premature and violent deaths, e.g. traffic accidents, having risky sexual intercourse [4,5] and poor academic performance, learning difficulties and school dropout [6-8]. In addition, heavy alcohol use during puberty appears to be related to damage to the development of cognitive and emotional abilities [9,10] and an elevated risk of later dependence and misuse [11,12]. Alcohol-related risks to cognitive functions seem to be higher in adolescents than in adults [11]. From the point of view of public health, prevention of heavy alcohol use among adolescents is essential.

There is little scientific evidence that universal prevention programmes aimed at youngsters affect drinking behaviour. Recent meta-analyses show that such programmes have small or no effects on alcohol use and binge drinking [3,13,14]. Exceptions to this are interventions aimed at both adolescents and their parents [15] and integrated programmes with multiple years of intervention and professional support [13,16,17]. Meta-analyses of school-based substance use prevention programmes have concluded that selective prevention programmes, targeting populations at increased risk, generally yield higher effects than universal programmes (e.g. [13,18]). According to Cuijpers and colleagues [13], selective prevention programmes have proved effective, but the availability of these programmes is limited. Therefore there is a recognized need in the field of substance use prevention for selective prevention programmes.

### Preventure

Preventure is a selective prevention programme and is one of the few school-based programmes with long-term effects on adolescents' drinking behaviour and binge drinking [16,19,20]. In research conducted in Canadian and English samples of adolescents, effects of the programme were found on abstinence, quantity and frequency of drinking, binge drinking, and problem drinking symptoms at four months and one year after the programme [16,19]. In addition to the effects on alcohol use, positive effects were found on emotional and behavioural problems, i.e. depression, panic attacks, truancy, and shoplifting [21].

The Preventure programme specifically targets young adolescents who have two well-known risk factors for heavy alcohol consumption: early-onset alcohol use [22,23] and personality risk for alcohol abuse (e.g. [24]). The programme is based on the theory that personality is an important construct for understanding adolescents' alcohol use and abuse. Two personality dimensions were previously found to be predictive of heavy alcohol use and alcohol use disorders, namely (1) an impulsive sensation seeking dimension, and (2) a behavioural inhibition dimension [16]. The first category involves young sensation seekers and young people with low impulse control, the second reflects a neurotic personality involving more anxious and negative thinking young people. Within these two dimensions, Conrod and colleagues [16] distinguished four personality profiles at higher risk of developing alcohol problems: Sensation Seeking (SS), Impulsivity (IMP), Anxiety Sensitivity (AS) and Negative Thinking (NT).

The four personality profiles were subsequently found to be strongly related to adolescents' quantity and frequency of drinking, frequency of binge drinking, and severity of alcohol problems [25,26]. Each personality profile is associated with specific substance misuse patterns, maladaptive motives for use, and vulnerability to specific forms of co-morbid psychopathology in adolescents [27,28]. Impulsivity is related to an increased risk of the early onset of alcohol and drug problems [29]. Sensation seekers drink more [30], tend to drink in order to enhance euphoric (intoxicating) effects [28], and are more at risk of adverse drinking outcomes (e.g. [30]). Highly anxiety sensitive persons show increased levels of drinking [31], are more responsive to the anxiety-reducing effect of alcohol, and are more likely to use alcohol to cope with negative feelings [28]. Persons with high levels of hopelessness often have depression-specific motives for alcohol use [32] and usually drink to cope with negative feelings [16,28,33,34].

The Preventure programme screens a school population for pupils who already drink alcohol and, additionally, belong to one of the four high-risk personality profiles. The programme identifies and treats high-risk adolescents, with the aim of preventing or intervening early before the high-risk adolescents engage in risky behaviours and/or these behaviours become problematic. The selected pupils are offered a tailored intervention based on cognitive behaviour therapy (CBT) and motivational interviewing. Cognitive behavioural techniques are used to target maladaptive thinking and coping skill deficits, and motivational interviewing techniques are used to address motivation to take responsibility for one's problematic behaviours. Motivational interviewing has proven to be effective for alcohol- and drug-related behaviour, and CBT can lead to reduction in anxiety sensitivity, depressive cognitions, and impulsivity (e.g. [35,36]). The manualized intervention, developed by Conrod and colleagues [35], provides personalized feedback and personality-specific cognitive-behavioural exercises designed to facilitate more adaptive coping. The focus is not on drinking (or drug use) per se but on risky ways of coping with personality, such as avoidance, distraction, and aggressive thinking, that may lead to substance misuse or other risky behaviour.

### Aims and hypotheses

In 2009, a project was started to develop and test Preventure in the Netherlands, where currently there is no selective school-based alcohol prevention available [37]. The main objective of this project is to study the effectiveness of Preventure on drinking behaviour of young adolescents in secondary education in the Netherlands. The effectiveness of the Dutch Preventure is being assessed by conducting a clustered randomized controlled trial (RCT), with two conditions (treatment and control arms). This is the first time that Preventure has been studied outside the setting where it was developed, England and Canada, to prove its effectiveness outside this setting.

The most relevant outcomes are percentage reductions in binge drinking (≥ five drinks on one occasion in the past four weeks), weekly drinking, and drinking-related problems after 2, 6, and 12 months. The main hypothesis is that high-risk students who receive the personality targeted intervention will score lower on these outcomes relative to those in the no-treatment control group. In addition, our secondary aim is to test the effects of the programme on emotional and behavioural problems (e.g. aggression, truancy, and shoplifting). Our hypothesis is that Preventure facilitates lower depression rates, lower anxiety rates, lower delinquent behaviour rates, less problem behaviour, and lower truancy.

## Methods/Design

### Study design and time frame

**Figure 1:**
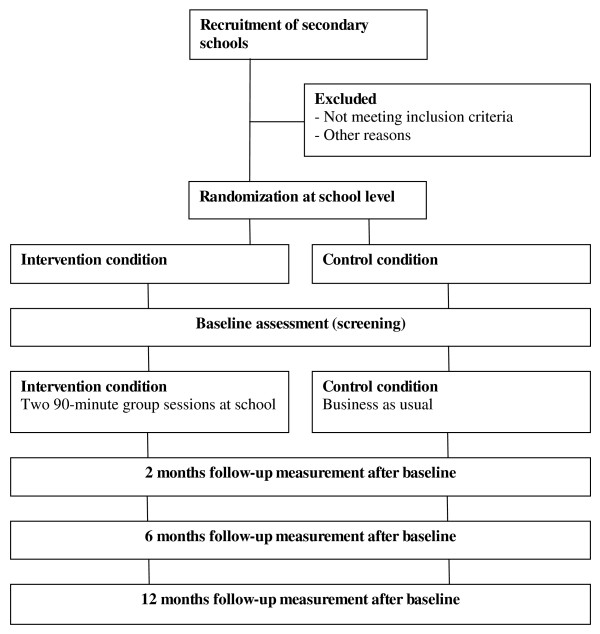
**Study design**.

The Preventure study is a 1-year RCT with two arms, an intervention and a control condition, testing the prevention programme effects, at 2, 6, and 12 months after the intervention (see Figure 1). Randomization is carried out at school level. The intervention condition consists of two group sessions based on cognitive behaviour therapy and motivational interviewing. The control condition receives no further intervention (business as usual).

The recruitment, inclusion, and randomization of the participants (schools and students) started in Spring 2009. The data collection started in 2010. The final follow-up measurement is planned for the end of 2011.

### Participants

#### Recruitment

A total of 100 schools were selected randomly from a list of all public secondary schools (N = 405) in four regions in the Netherlands (Zuid-Holland, Utrecht, Gelderland, Overijssel). Schools were invited to participate in the study, if the following inclusion criteria were met: 1. school had at least 600 students, 2. < 25% of students were from migrant populations, 3. school did not offer special education. A total of 15 schools were willing to participate and fulfilled the inclusion criteria. The main reasons for schools not participating were lack of time and no interest in participating in research in general.

#### Eligibility

Students were eligible to enter the trial if they fulfilled the following inclusion criteria: 1. life time prevalence of alcohol use (i.e. having drunk at least one glass of alcohol once in their life), 2. belonging to one of the four personality high-risk groups for (future) heavy drinking (AS, SS, NT or IMP) and 3. informed consent of the student and his or her parents. The study is aimed at students from 13 to 15 years of age. This is in contrast to Conrod et al.'s study [16], in which students aged 14 to 17 were studied. The reason for this difference is the age of onset, which is lower among Dutch youngsters than among their study sample.

In order to select those students fulfilling the selection criteria, a screening survey among all students attending grade 8 and grade 9 of the 15 schools was carried out. The students who scored more than one standard deviation above the sample mean on one of the four personality risk scales (AS, SS, NT, or IMP) of the Substance Use Risk Profile Scale (SURPS) [25], were classified as belonging to a risk group for the development of alcohol problems. If a student scored high on more than one subscale, he or she was assigned to the personality group in which he or she showed the largest statistical deviation with respect to the z-scores.

#### Consent

Parents were informed of the study (screening and intervention) through a letter sent home from the schools asking them to contact the researchers by phone or e-mail if they did not wish their child to participate in the study (passive informed consent). Parents were told that the intervention was coping-skill training designed to reduce adolescent risk taking, with alcohol abuse as an example. To assure participants' confidentiality, parents were not explicitly informed about any of the selection variables of the study. On the day of the screening, students were given information on the screening, the ethical issues (confidentiality and the voluntary nature of participation), and the intervention. Parents and students provided active informed consent to participate in the intervention part of the study.

The study was evaluated by the Medical Ethical Commission for Mental Health (METIGG), which considered the study did not fall within the WMO Act (Medical Research Involving Human Subject Act). As a result no ethical approval was necessary. However, for the consent procedure, we adhered to the guidelines and advices of the METIGG.

#### Randomization

Randomization occurred at the school level to avoid contamination between conditions. An independent statistician assigned the participating schools randomly to one of the two conditions: intervention or control. Randomization was carried out using a randomization scheme, stratified by level of education and school size, with the schools as units of randomization.

### Sample size

#### Power

The power calculation reflects the idea that we want to induce a reduction in the percentage of students engaging in binge drinking (drinking five or more glasses of alcohol on one occasion) at least once during the last four weeks, from the current estimated 50% (among life-time users grade 9/10; estimate based on the results of a national school survey, [1]) to 35%. For a 15% reduction after 12 months among the students in grade 9/10, a sample size of N = 183 in each condition was required to test the hypothesis in a 2-sided test at alpha = 0.05 and a power of (1-beta) = 0.80. Because of the loss of power due to randomization of schools (and not students) and the increase in error because of applying a multiple imputation procedure to fill in missing values, 183*1.4 = 256 respondents per condition (intervention and control) needed to be included at baseline to test the effectiveness of the Dutch Preventure programme.

#### Number of students

According to the power analyses, a net sample of 256 respondents in each condition was needed. On the assumption of a 40% participation rate, 45% of respondents belonging to one of the risk groups (estimates based on [16,19]), a life time prevalence at baseline of 77%, and 93% of children present in the class at the data collection time (estimates based on [1]), a survey sample of N = 3,972 students was needed.

### Study intervention

To develop the Dutch Preventure programme, the principles and guidelines of the original Canada/UK programme were followed in collaboration with the original developers of Preventure.

#### Theoretical basis

Preventure incorporates the principles from motivational and cognitive behavioural therapy and is adapted to different personality profiles for substance abuse: anxiety sensitivity, negative thinking, sensation seeking, and impulsivity. The intervention is brief, as the literature strongly suggests that brief interventions can be very effective in changing drinking patterns and related problems. An effective component of successful brief interventions for alcohol abuse is the persuasiveness of individualized feedback. Therefore, Preventure provides pupils with personalized feedback on their results from a personality and motivational assessment. Preventure also includes cognitive behavioural skills training specifically relevant to each personality profile. The literature has shown that successful cognitive behavioural therapy can lead to reductions in anxiety sensitivity in anxiety patients, depressive cognitions in depressed patients, and impulsivity in adolescents with externalizing disorders [38,39,36].

The intervention consists of three main components: (1) psycho-education, (2) behavioural coping skills, and (3) cognitive coping skills [16]. In the coping skills sections, students are engaged in activities to induce automatic thoughts. Simultaneously, they are trained to use cognitive restructuring techniques to counter such thoughts. Cognitive restructuring training has been shown to have a positive impact on the reduction of alcohol and drug abuse and symptoms of psychological disorders [35].

#### Intervention condition

The intervention involved two group sessions, carried out at the participants' schools. The group sessions were adapted to one of the four personality profiles. This means that there were four different groups of two sessions each. Both group sessions lasted 90 minutes and were spread across two weeks. The intervention was provided by a qualified counsellor and a co-facilitator. The three counsellors and two co-facilitators had received two days training from Dr. P.J. Conrod, who developed the original intervention. Furthermore, all the counsellors had practiced the two group sessions at a pilot school with students who met the inclusion criteria (drinkers with high-risk personality profiles).

The intervention used student manuals. The original student manuals, developed in Canada, were translated and adapted to the cultural and school context of the Netherlands. The examples, the real-life stories, and the illustrations used in the programme manuals were adapted to the Dutch situation. The student manuals consist of text, exercises, and real-life experiences or scenarios. The real-life scenarios were generated by previously organized focus groups of high-risk personality adolescents. In four focus groups (one group for each personality risk factor), students were asked to share their own experiences regarding, for example, alcohol and drugs. The student manuals had been tested during the pilot sessions at the pilot school. Students were asked to give their opinion on the content, the illustrations, and real-life stories used in the manuals.

In the first group session, psycho-educational strategies were used to educate students about the target personality variable (NT, AS, IMP, or SS) and the associated problematic coping behaviours, such as interpersonal dependence, aggression, risky behaviours, and substance misuse. Students were motivated to explore their personality and ways of coping with their personality through a goal-setting exercise. Thereafter, they were introduced to the cognitive behavioural model by analysing a personal experience according to the physical, cognitive, and behavioural responses.

In the second session, participants were encouraged to identify and challenge personality-specific cognitive thoughts that lead to problematic behaviours. For example, the impulsivity intervention focused on not thinking things through and aggressive thinking, and the sensation-seeking intervention focused on challenging cognitive thoughts associated with reward seeking and boredom susceptibility.

#### Control condition

Students assigned to the control group received no further intervention. An inventory among the participating schools will reveal whether other specific substance use prevention programmes were being used, apart from the common lessons in the curriculum, e.g. biology classes.

### Data collection and instruments

The screening survey collected data by means of an online questionnaire on alcohol use, demographics, and personality risk factors. The data collection took place during a regular lesson (approximately 50 minutes), and questionnaires were administered by a research assistant from the Trimbos Institute. Those students randomly assigned to the experimental or control condition have completed, or will complete, the post-treatment survey after 2, 6, and 12 months. Data for the follow-up measurements have been, or will be, also collected online at school. The follow-up survey contains the same assessments as the screening survey. An overview of all measurements is given in Table 1.

**Table 1:** Overview of measurements

Measurement	Baseline (screening)	Follow-up I (2 months after baseline)	Follow-up II (6 months after baseline)	Follow-up III (12 months after baseline)
Demographic characteristics	*	*	*	*
Truancy	*	*	*	*
Alcohol:				
Drinking behaviour	*	*	*	*
Drinking motives	*	*	*	*
Drinking problems	*	*	*	*
Perceived parental rules	*	*	*	*
Drinking parents	*	*	*	*
Tobacco:	*	*	*	*
Smoking behavior	*	*	*	*
Smoking parents	*	*	*	*
Perceived parental rules	*	*	*	*
Marijuana:	*	*	*	*
Marijuana-using behaviour	*	*	*	*
Marijuana parents	*	*	*	*
Other:	*	*	*	*
Personality	*	*	*	*
Anxiety	*	*	*	*
Psychological problems	*	*	*	*
Delinquency	*	*	*	*
Depression	*	*	*	*
Self control	*	*		

As already mentioned, the SURPS [25] distinguishes four personality profiles. Each profile is assessed using five to seven items that could be answered on a 4-point scale, 1 = *strongly disagree*, 2 = *disagree*, 3 = *agree*, 4 = *strongly agree*. The SURPS scale has 23 non-overlapping items that assist in discriminating personality dimensions independent of substance use behaviour. Negative Thinking (7 items) refers to hopelessness, which might lead to depressive symptoms. A sample item on the Negative Thinking subscale is 'I feel that I'm a failure.' The Anxiety Sensitivity dimension (5 items) measures fear of bodily sensations, and an example item is 'It frightens me when I feel my heart beat change.' The Sensation Seeking subscale (6 items) measures the tendency to seek out thrilling experiences, e.g. 'I would like to learn how to drive a motorcycle.' The tendency to act without thinking is measured by the Impulsivity subscale (5 items), and an example of this subscale is 'I often don't think things through before I speak.' Studies in both adolescent and adult samples in several countries, including the Netherlands, have shown that this scale has good internal reliability, good convergent and discriminant validity, and adequate test-retest reliability [34,40,41,25,27]. The instrument was translated into Dutch by an English speaking language consultant, has been successfully applied [34], and was tested at schools before use in the screening survey.

### Outcomes

When the data analysis takes place, the primary outcomes will be percentage reductions in binge drinking, weekly and weekend drinking, and drinking-related problems. To assess life-time alcohol use and binge drinking, two questions will be used that are widely used in school surveys, including the ESPAD study [2], Monitoring the Future [42], and the national school surveys in the Netherlands [1,43]. The average standard units in the last week will be assessed with the Weekly Recall [44,45]. Weekly and weekend alcohol use is defined by the quantity-frequency measure [46,47]. To assess behavioural symptoms of adolescent problem drinking, the Rutgers Alcohol Problems Index (RAPI) [48] will be used. The RAPI has been well validated for use with both clinical and community adolescent samples [49-51,48].

Other outcomes will include percentage reductions in depressive feelings, anxiety symptoms, problem behaviour, drinking motives, truancy, and delinquent behaviour. Depressive feelings will be measured with the widely used 20-item (Dutch version) of the Centre for Epidemiological Studies Depression Scale (CES-D) [52,53]. The Childhood Anxiety Sensitivity Index (CASI) [54] is a self-report questionnaire to assess children's and adolescents' fear of anxiety symptoms. The CASI has good internal consistency and acceptable 2-week test-retest reliability [55]. The Strengths and Difficulties Questionnaire (SDQ) [56] will be used as a behavioural screening instrument for early detection of psychological problems. The DMQ-R [57] is the most widely used instrument to assess drinking motives among young people. The DMQ-R has been well validated in several international (e.g. [58]) and national studies (e.g. [51]).

### Statistical Analyses

Descriptive analyses will be conducted to examine whether randomization resulted in a balanced distribution of important demographic characteristics and the outcome variables in the two conditions. To control for potential bias, possible confounders will be included in all further analyses.

Analyses will be performed according to the intention-to-treat and completers-only principles, controlling for sex, age, and educational level. Intention-to-treat means that all participants will be analysed in the condition to which they were assigned by randomization. Therefore, missing data at follow up will be imputed using regression imputation. With respect to the completers-only analyses, only the participants with scores on all time points will be included, without the inclusion of imputed data. In both the intention-to-treat and the completers-only analyses, the effects of the intervention condition will be compared with those of the control condition. For continuous outcome measures, t-tests, or Man Whitney U if non-parametric distributions, will be performed. When correction for confounding variables is necessary, multivariate regression analyses will be performed. The fact that the data are clustered, because groups of respondents that are attending the same class and/or school are investigated, will be taken into account in the analyses.

## Discussion

The present study protocol presents the design of a randomized controlled trial evaluating the effectiveness of a prevention programme called Preventure. The intervention programme aims to prevent adolescents from (problematic) alcohol drinking. It is hypothesized that, after one year of follow-up, students in the intervention condition will be engaging less in binge drinking and weekly drinking, and will have fewer drinking-related problems than those students in the control condition.

### Strengths and limitations

A first strength of Preventure is that it is one of the few school-based programmes with proven effects on drinking behaviour of adolescents [16,20]. Second, the programme is a selective prevention programme. In the field of substance use prevention in The Netherlands, there is a recognized need for selective prevention programmes [13]. Third, the intervention incorporates elements of motivational and cognitive behavioural theory, which have been proven to be effective in reducing alcohol abuse and associated psychological problems. Fourth, Preventure is a short intervention (two sessions), which makes it less time-consuming than regular prevention programmes and therefore easier to implement in schools. A limitation of the study is that the information on the behaviour of the adolescents is based on self-reports, which might lead to measurements errors. However, studies have shown that self-report data of adolescents about their own drinking, smoking, and drug use are generally reliable (e.g. [59,60]).

A general issue with targeted interventions is the selection of participants and providing information to the participants and their parents in an accurate manner. In this study, neither the parents nor the teachers at the school were explicitly informed about the selection variables of the study, to avoid stigmatization of the students. This ethical issue should also be taken into account if the programme is implemented at other schools in the Netherlands in the future.

### Implications for practice

If the Preventure prevention programme is effective, it can be implemented widely in schools in The Netherlands - for example, as part of the Dutch national school prevention programme The Healthy School and Drugs. The Healthy School and Drugs has a large network among institutions for care and treatment of drug addicts and schools.

## Conclusion

This study has described a programme, currently on trial in the Netherlands, for preventing and reducing binge drinking in adolescence. Evaluation of the programme will provide insight into the effectiveness of Preventure in the Netherlands and the precursors of alcohol use among Dutch adolescents.

## Competing interests

Patricia Conrod is one of the developers of the Preventure programme and is the principal investigator of the Preventure trials in the UK. The other authors declare that they have no competing interests.

## Authors' contributions

JL, FG, and SL are responsible for data collection, data analysis, and reporting the study results. PC is the principal investigator of the Preventure trials in the UK and Canada. MK, RE, and RW are supervisors. KM and LL have contributed to the grant. All authors read and approved the final manuscript.

## Pre-publication history

The pre-publication history for this paper can be accessed here:

http://www.biomedcentral.com/1471-2458/11/126/prepub
